# An evaluation of LLIN ownership, access, and use during the Magude project in southern Mozambique

**DOI:** 10.1371/journal.pone.0282209

**Published:** 2023-03-27

**Authors:** Lucia Fernández Montoya, Celso Alafo, Helena Martí-Soler, Mara Máquina, Arlindo Malheia, Charfudin Sacoor, Ana Paula Abílio, Dulcisaria Marrenjo, Nelson Cuamba, Beatriz Galatas, Pedro Aide, Francisco Saúte, Krijn P. Paaijmans

**Affiliations:** 1 Centro de Investigação em Saúde de Manhiça (CISM), Fundação Manhiça, Manhiça Maputo, Mozambique; 2 ISGlobal, Barcelona, Spain; 3 Instituto Nacional da Saúde, Ministério da Saúde, Maputo, Mozambique; 4 Programa Nacional de Controlo da Malária, Ministério da Saúde, Maputo, Mozambique; 5 PMI VectorLink Project, Abt Associates Inc., Maputo, Mozambique; 6 Center for Evolution and Medicine, School of Life Sciences, Arizona State University, Tempe, AZ, United States of America; 7 The Biodesign Center for Immunotherapy, Vaccines and Virotherapy, Arizona State University, Tempe, AZ, United States of America; 8 Simon A. Levin Mathematical, Computational and Modeling Sciences Center, Arizona State University, Tempe, AZ, United States of America; Swiss Tropical and Public Health Institute, SWITZERLAND

## Abstract

The Magude Project assessed the feasibly of eliminating malaria in a low transmission setting in southern Mozambique using a package of interventions. This study measured the ownership, access and use of long-lasting insecticide treated nets (LLINs) and inequalities in these indicators across household wealth, size and population subgroups, to understand the protection that LLINs provided during the project. Data were obtained from various household surveys. At least 31% of the nets distributed during the 2014 and 2017 campaigns were lost during the first year post-distribution. Most nets (77.1%) present in the district were Olyset Nets. LLIN access never exceeded 76.3% and use varied seasonally between 40% and 76.4%. LLIN access limited LLIN use during the project, especially during the high transmission season. LLIN ownership, access and use were lower in harder-to-reach localities, in poorer and larger households. Children and women below 30 had poorer access to LLINs than the overall population. Net use was lowest among school-aged children and young adults, especially among young males, and highest in children under 5, pregnant women, in older adults and in households that received indoor residual spraying (IRS). This study revealed that LLIN mass-distribution campaigns alone are not sufficient to achieve the high level of net protection needed during elimination programs and that reviewing the LLIN allocation scheme, top-up distributions and/or community engagement campaigns is needed, also to reduce inequalities in populations’ access to LLINs.

## Introduction

Mozambique is one of the six countries that accounts for approximately half of all malaria deaths worldwide [1]. Although most of the country-wide efforts concentrate on reducing the malaria burden, malaria eliminating initiatives have been implemented in the southern part of the country in the context of broader attempts to eliminate malaria in southern Africa [2, 3]. In Mozambique, both control and elimination efforts have relied heavily on vector control. Until 2000 malaria prevention relied mainly on indoor residual spraying. Since 2000 it relied on the distribution of insecticide-treated nets (ITNs), later replaced by long-lasting insecticidal nets (LLINs), while the use of indoor residual spraying (IRS) has been limited to a few districts in Central Mozambique and to the southern province of Maputo to accelerate malaria elimination in this region, South Africa and Eswatini [2, 3]. LLINs have been used as a core vector control intervention since 2005, first distributed to target groups and then through mass distribution campaigns every 3 years since 2014, with the aim to provide at least one net for every two persons in a household [4].

LLIN efficacy relies on their ability to kill mosquitoes when they come into contact with the net, and their ability to prevent vectors from biting humans (as they provide a physical barrier and/or repel host-seeking mosquitoes). LLIN effective protection in the field depends on net use, which depends on a population’s access to LLINs [5]. Once in use, net effectiveness depends on its physical integrity (absence of holes), ability to preserve the bio-availability of insecticides on the net’s surface [6, 7] and on the behavior of local vector populations [8], as LLINs cannot prevent bites from vectors that bite outdoors, or indoors before people use a net. Evidence from the field shows that LLIN ownership, access, physical integrity and residual bio-efficacy can all decrease rapidly over time after net distribution [9–11], that LLIN use can vary markedly across seasons [12] and that vector compositions and behaviors can change as a results of the implementation of vector control interventions [13, 14].

LLINs were one of the two vector control interventions implemented during the Magude project. The project aimed to interrupt malaria transmission in Magude district, southern Mozambique, through a combination of interventions targeting the vector (LLINs and annual district-wide indoor residual spraying or IRS) and the parasite reservoir (mass drug administration, MDA, and standard diagnosis and treatment) simultaneously [15], but local malaria transmission was not halted [15, 16].

This study aimed to evaluate the extent of protection that was provided by LLINs during the Magude project, to better understand why malaria transmission could not be interrupted locally. Results from this study also inform the design of additional strategies to cover the gaps in LLIN protection in future malaria elimination efforts in the region. Using data collected through the district demographic and health platform, malaria prevalence cross-sectional surveys, and mass drug administration campaigns, we evaluate LLIN ownership, access, and use during the Magude project, as well as differences in these indicators across district localities, household wealth and size, age groups and gender.

## Materials and methods

### Study site and LLIN distribution

The study was conducted in Magude district, southern Mozambique. A baseline census in 2015 registered 48,448 residents (and 4,133 non-residents) and 10,965 households. Additional detailed demographic, health and malaria incidence information can be found elsewhere [17]. Magude, like most of Mozambique, has year-round malaria transmission with seasonal variations presenting higher incidence between November and April. Two LLIN mass distribution campaigns were conducted by the National Malaria Control Programme (NMCP), one before (May 2014) and one during the Magude project (December 2017). In May 2014, 35,432 LLINs (Olyset, Sumitomo Chemical Ltd, Japan; Permanet 2.0, Vestergaard Frandsen, Switzerland) were distributed in the district [17], and in December 2017, 44,400 LLINs (Dawa Plus 2.0, Tana Netting, United Arab Emirates). Additionally, LLINs were continuously distributed through the Expanded Program of Immunization (EPI) and antenatal care services (ANC), although the total number of nets and net brand(s) distributed through these channels are unknown. Community engagement activities were conducted throughout the Magude project to increase the acceptability of MDA and IRS and the use of LLINs.

### Data sources

The present analysis draws from data collected through multiple studies and surveys conducted throughout the Magude project. A summary of each study with its sampling strategy is provided below. More extensive description of the malaria prevalence cross sectional surveys, mass drug administration surveys, the census of the population and demographic and health surveys are provided elsewhere [15–17]. Table 1 shows the LLIN indicators that were monitored, as well as the sample size of each study.

**Table 1:** Data sources, their sample sizes and LLIN indicators estimates derived from each source.

	2015			2016				2017		2018	
Time	Jan-June	May	Nov	Jan	May	Jun-Aug 16	Dec	Feb	May	May	Sep-Dec
Type of study	Population census	Prevalence Survey	MDA 1	MDA 2	Prevalence Survey	HDS 1	MDA 3	MDA 4	Prevalence Survey	Prevalence Survey	HDS 2
Sample size	10800 households, 48448 people 24302 nets	1035 people	43431 people interviewed	37666 people interviewed	1657 people (age stratified sample)	10648 households, 49274 people	39759 people interviewed	39748 people interviewed	3865 people (age stratified sample)	3354 people (age stratified sample)	10149 households, 51436 people
LLIN channels of acquisition											
LLIN brands											
LLIN attrition since last campaign											
LLIN ownership											
LLIN access											
LLIN use											
LLIN sharing: no of people that slept under each net											
LLIN reasons for not use											

Prevalence Survey: Malaria prevalence cross sectional surveys

MDA: Mass drug administration campaigns

HDS: Health and demographic surveys

- A **census of the population** (January-June 2015) was conducted before the Magude project began. The questionnaire included questions at the household, individual and net levels, including the number of members and nets that each household had, the net brands, the number of people that slept under each net and whether people used the net the night before the survey. In the individuals’ surveys, only residents’ answers were recorded (i.e. visitors were excluded) [17].
- **Health and demographic surveys** (2 surveys; August-September 2016 and September-December 2018) were conducted to update a subset of data that were collected during the census of the population and included the entire Magude population. The questionnaire included questions on number of household members and nets in each household and whether people used the net the night before the survey, but did not record the net brand nor the number of people that slept under each net [17].
- **Malaria prevalence cross-sectional surveys** (4 surveys, every May from 2015 to 2018) were conducted to measure malaria prevalence at the end of the high transmission season. An age-stratified simple random sample of participants, with oversampling of children under 15 years of age, was drawn from the census of the population annually. Each participant was asked about the type of net they slept under, the channel of net acquisition and whether they slept under the net the night before the interview.
- **Mass drug administration surveys** (4 surveys at each MDA round: November 2015, January 2016, December 2016 and February 2017) were administered to each person found during each MDA round, regardless of whether they received the drug or not. The percentage of the population that responded to these surveys was 89.6% in November 2015, 77.7% in January 2016, and 80.7% in both December 2016 and February 2017. The questionnaire included questions on whether people used the net the night before the survey, and the reason for not using it.

Questionnaire data (interviews) were collected using tablets and Open Data Kit (ODK [18]) forms and sent daily to a Server Data Base at CISM (Manhiça Health Research Center, Manhiça, Mozambique).

### Data analysis

#### LLIN attrition since the 2014 mass distribution campaign

LLIN attrition was calculated retrospectively, using data from the 2015 population census and from the health and demographic surveys in 2016 and 2017. The number of nets present in households was compared with the number of nets distributed during the mass distribution campaign to estimate the minimum percentage of nets lost.

#### LLIN ownership, access and use and inequalities across household and population subgroups

Bednet ownership, access and use indicators were estimated following the recommendations of Roll Back Malaria and the World Health Organization [19, 20].

- Ownership (calculated from census data and health and demographic surveys):
  ○ proportion of households with at least one LLIN out of all households in the district.
  ○ proportion of households with at least one LLIN for every two persons out of all households in the district (referred here to as “optimal ownership”).
- Access (calculated from census data and health and demographic surveys):
  ○ proportion of individuals with access to a net in their household out of all individuals in the district.
  ○ proportion of individuals sleeping in households that had at least one net for every two people out of all individuals in the district.
- Use (calculated from census data and health and demographic surveys, MDA surveys and malaria cross-sectional prevalence surveys):
  ○ proportion of individuals that slept under a net the night before being interviewed.

To understand inequalities in LLIN ownership and access across localities and types of households, the above indicators were calculated for each of the five administrative posts, each level of household wealth and each household size (i.e. the number of members in a household). Household wealth was calculated using the Multidimensional Poverty Index (PI), an adaptation of the poverty index originally developed by the Oxford Poverty and Human Development Initiative [21], using data from the Magude census of the population and by classifying the household into three groups according to their deprivation scores: 0–2; 3–4; 5–6. The higher the number of deprivations, the poorer the household. A complete explanation of the calculation of the Multidimensional Poverty Index (PI) is provided elsewhere [22].

Differences in LLIN ownership and access across households of different deprivation levels were calculated using data from the 2015 census, as this was the only survey with sufficient data to estimate the Multidimensional Poverty Index (PI). Differences across household size and administrative post were calculated based on pooled data from the 2015 census and the health and demographic surveys in 2016 and 2018. To explore differences in net access across sex and age groups, we calculated the percentage of people living in households with at least one net for every two people, disaggregated by sex and by age group (i.e. under 5 years of age, between 5 and 14 years of age and 15 years of age) for each survey, and statistical analyses were performed using the Chi-square test of inequality.

To estimate LLIN use from the malaria prevalence cross-sectional surveys, weights were assigned to participants’ answers based on their age strata as the random sample was age-stratified. Resulting point estimates are provided together with their 95% confidence intervals (CIs) using the svycipro function of the R package survey. To estimate LLIN use from MDA surveys, point estimates together with the ‘best-case’ and ‘worst-case’ scenario are provided, as those surveys did not reach the entire population (they reached between 76% and 80% of the population). For the best-case scenario, we assume all individuals who were missed slept under the net the night before the survey. For the worst-case scenario, we assumed none of those individuals slept under a net.

Additional analyses were conducted using the census and the health and demographic surveys to understand i) the behavioral gap in LLIN use (i.e., the percentage of people that used the net of those living in a household with at least one LLIN for every two people, disaggregated by sex and the age groups; ii) if and how the population shared the net (i.e. calculation of the percentage of nets that were shared by one, two or three or more people) in households with different numbers of people per net, and iii) the effect of IRS on LLIN use (i.e. the percentage of people sleeping under a net in sprayed vs. unsprayed household).

#### Reasons for not using an LLIN to sleep

In surveys conducted during the MDA campaigns (high transmission seasons), participants were asked for the reason for not using a net to sleep the night before, through a closed-ended question. During the MDA surveys the following options were provided: I don’t have a net; I dislike the net; the net was not hung; it is too hot; other reason (please specify). The percentage of individuals reporting each answer is reported together with 95% Wald Cis. Estimates represent between 76% and 80% of the Magude population, depending on the MDA survey round.

#### Type of nets used by residents and channels of net acquisition

We report the frequency of each net brand found in the district during the 2015 population census. Malaria prevalence cross-sectional survey data (2015, 2016, 2017, 2018) were used to estimate the percentage of participants that slept under an LLIN or a net impregnated in the last 12 months and the frequency of different channels of acquisition (distributed by a health facility, by the NMCP, by the Centro de Investigação em Saúde de Manhiça, bought or unknown). Weights were assigned to participants’ answers based on their age strata (see above) and analyses were performed using the svycipro function of the R package survey.

### Ethical consideration

All studies were approved by CISM’s institutional ethics committee, Hospital Clinic of Barcelona’s Ethics Committee, and the Mozambican Ministry of Health National Bioethics Committee. The study protocol to implement and evaluate the impact of MDAs was also approved by the pharmaceutical department of the MoH of Mozambique and registered as Clinical Trial NCT02914145. More details on the ethical considerations of the population census, household surveys, cross-sectional surveys and MDAs are provided elsewhere [16]. Written informed consent and assent (for 12 to 17 year olds) was sought from all individuals, or parents/guardians if participants were younger than 18 years old, who participated in each study before conducting any study procedures.

## Results

### LLIN attrition since mass distribution campaigns

In Jan-Jun 2015, less than one year after the 2014 mass distribution campaign, the total number of nets in the district (including those not distributed during the mass campaign of 2014) was 25,011 [17]. This number was 22,502 in June-August 2016 and 30,274 in September-December 2018. This implies that at least 31.4% and 36.5% of the nets that were distributed during the 2014 mass distribution campaign were lost during the first and second year after distribution, respectively, and that 31.8% of the nets distributed during the 2017 mass campaign were lost within a year of distribution.

### LLIN ownership and access

The proportion of households that owned at least one net decreased from 81.5% in 2015 (Jan-June) to 78.8% in 2016 (Aug-Sept) but increased to 91.1% in 2018 (Sep-Dec) after the mass distribution campaign of December 2017. The proportion of households that owned at least one net for every two persons decreased from 61.9% in 2015 to 54.4% in 2016 and increased again to 59.2% in 2018. The proportion of individuals that had access to an LLIN within their household was 73.7% in 2015, decreased to 68.2% in 2016 and increased to 76.3% in 2018. A summary of net ownership and access values obtained during the population census and subsequent district-wide health and demographic surveys is provided in Table 2.

**Table 2:** LLIN ownership, access, use, use provided access and use among sprayed and unsprayed household throughout the Magude project. Indicators estimated from MDA surveys are presented with their best-case and worst-case estimates in parenthesis. Confidence intervals for indicators estimated from the malaria prevalence cross-sectional surveys are calculated with survey methods taking into consideration individual weights based on their age-strata and are presented in square brackets. No confidence intervals are provided for indicators calculated from the census or demographic and health surveys, as these covered the entire population in the district.

			2015			2016				2017		2018	
			Population census	Prevalence Survey	MDA 1	MDA 2	Prevalence Survey	HDS 1	MDA 3	MDA 4	Prevalence Survey	Prevalence Survey	HDS 2
			Jan-June	May	Nov	Jan	May	Jun-Aug 16	Dec	Feb	May	May	Sep-Dec
**Ownership**	% of households with at least one LLIN		81.5					78.8					91.1
	% of households with at least one LLIN for every two people		61.9					54.4					59.2
**Access**	% of individuals with access to an LLIN in their household		73.7					68.2					76.3
	% of individuals who lived in households with at least one net per every two people		54.5					45.6					50.0
	% of children <5 years-old who lived in households with at least one net per every two people		48.9					39.9					45.2
	% of pregnant women who lived in households with at least one net per every two people							49.6					50.8
**Individual level use**	% individuals who slept under an LLIN the previous night	Overall	25.4	40.9 [36.7–45.0]	67.9 (61.0, 75.2)	76.3 (59.4, 81.7)	64.4 [61.6-67-0]	40.00	67.8 (54.7, 74)	70.3 (56.7, 76)	72.2 [70.0–74.0]	70.4 [67.7–73]	57.1
		In HH with at least one net for every two people	35.0					55.1					66.1
		In HH with less than one net for every two people	14.0					27.6					49.2
	% of < 5 year-olds who slept under an LLIN the previous night	Overall	27.2	39.5 [35.4–44.0]	73.6 (67.9, 75.6)	78.8 (61.8, 83.4)	68.6 [63.9–73.0]	43.8	71.9 (65.3, 74.5)	74.4 (68.1, 76.5)	80.8 [79.0–82.0]	76.7 [74.4–79]	64.6
		In HH with at least one net for every two people	36.7					59.5					74.1
		In HH with less than one net for every two people	18.0					34.0					58.2
	% of 5–14 year-olds who slept under an LLIN the previous night	Overall	21.7	40.3 [33.8–47]	69.8 (52.3, 77.4)	75.6 (56.7, 81.6)	60.5 [54.7–66.0]	36.5	65.2 (52.6, 71.9)	68.2 (54.2, 74.8)	70.3 [67.5–73.0]	70.7 [68.3–73.0]	58.3
		In HH with at least one net for every two people	31.0					52.2					67.5
		In HH with less than one net for every two people	11.1					25.0					50.5
	% of >15 year-old who slept under an LLIN the previous night	Overall	26.9	41.7 [35.0–49.0]	70.8 (56.8, 76.6)	76.0 (58.7, 81.5)	65.4 [61.5–69]	40.9	68.0 (52.8, 75.2)	70.1 (54.8, 76.6)	70.6 [67.0–74.0]	68.0 [63.2–72.0]	54.6
		In HH with at least one net for every two people	36.7					55.5					63.7
		In HH with less than one net for every two people	14.2					26.9					45.9
	% of men who slept under an LLIN the previous night	Overall	24.0	34.8 [29.0–41.0]	69.9 (55.2, 76.2)	75.4 (52.8, 82.8)	63.5 [63.5–68]	38.5	66.1 (50.2, 74.3)	68.4 (52.3, 75.9)	72.4 [69.1–75.0]	69.4 [65.4–73.0]	53.0
		In HH with at least one net for every two people	33.3					53.8					62.4
		In HH with less than one net for every two people	13.2					26.5					46.1
	% of women who slept under an LLIN the previous night	Overall	26.6	45.5 [39.6–51.0]	71.9 (65.7, 74.3)	77.1 (64.7, 80.8)	65.0 [61.4–68.0]	41.2	69.0 (58.3, 73.9)	71.6 (60.1, 76.2)	72.3 [69.5–75.0]	71.0 [67.4–74.0]	60.2
		In HH with at least one net for every two people	36.4					56.0					69.1
		In HH with less than one net for every two people	14.6					28.4					51.9
	% of pregnant women who slept under a net the previous night	Overall		42.9 [9–85]	73.1^a^	77.9^b^	77.8 [50.5–92]	48.6	72.0^c^	77.9^c^	90.0 [64.4–98.0]	92.3 [53.1–99.0]	62.8
		In HH with at least one net for every two people						59.7					70.9
		In HH with less than one net for every two people						38.8					55.6
**Relation between LLIN use and IRS**	% individuals who slept under an LLIN the previous night	In sprayed HH	27.0	43.1 [37.8–49]	73.0^a^	78.7^b^	67.5 [64.4–70.0]	41.7	69.4^c^	71.5^c^	71.6 [68.9–74.0]	73.2 [69.8–76.0]	
		In unsprayed HH	23.2	37.1 [30.4–44]	60.7^a^	66.4^b^	48.5 [40.8–56.0]	37.7	56.0^c^	59.5^c^	67.6 [62.0–73.0]	60.8 [54.5–67.0]	

HH = household, HDS = Health and demographic survey, a: based on answers from 89.6% of the district residents, b: based on answers from 77.7% of the district residents, c: based on answers from 80.7% of the population

**Fig 1:**
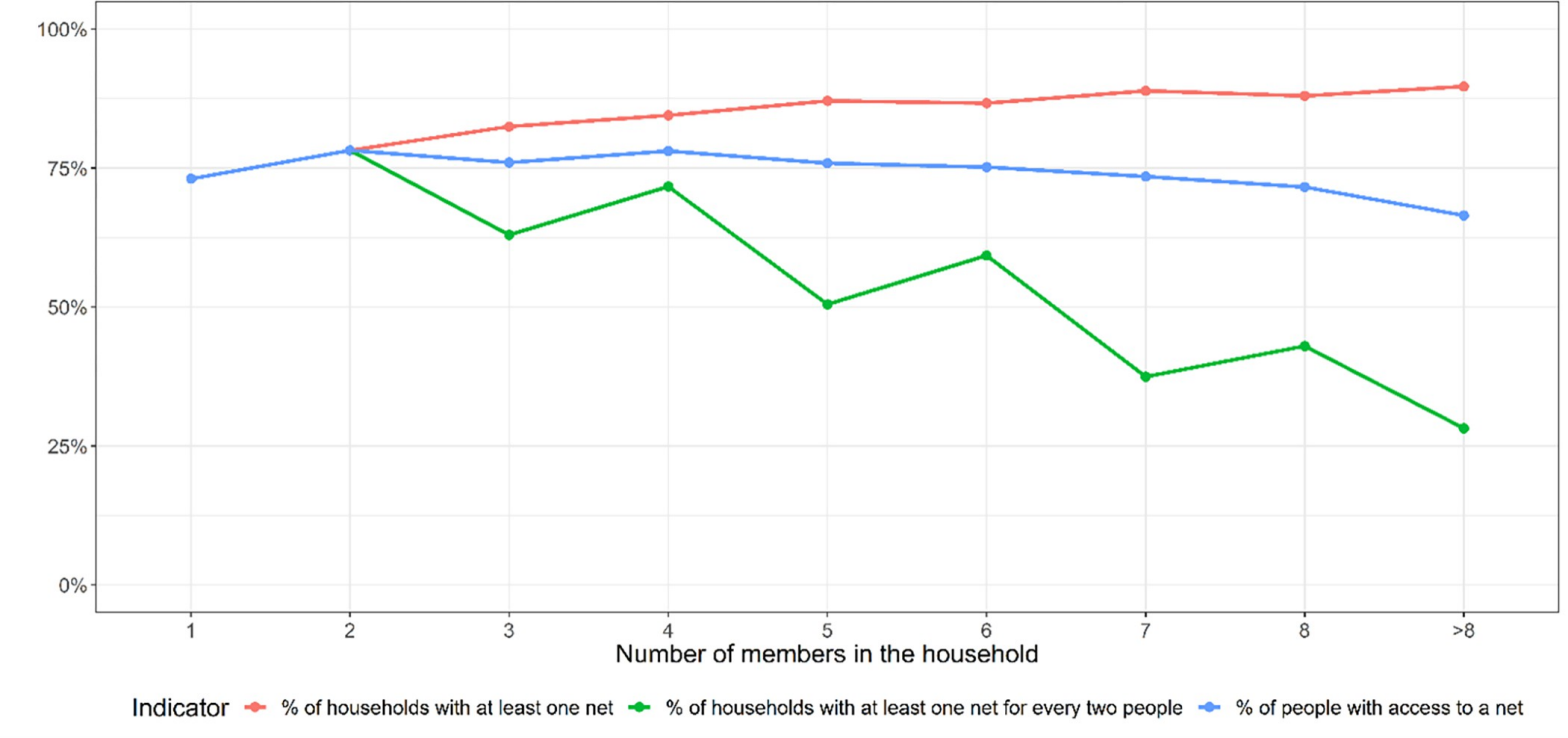
Percentage of households in Magude district with at least one LLIN, of households with a least one LLIN for every two people, and of household members with access to a net, segregated by the size of the households.

There were significant differences in ownership and LLIN access across household sizes (χ^2^, df = 8, p<0.0001 for all tests). The percentage of households with at least one LLIN increased slightly with increasing household size (i.e. number of members in the household), but the percentage of households with at least one net for every two people (optimal ownership) and the percentage of people with access to a net decreased with increasing household size. The percentage of households with at least one net was 73.1% for households with one person and 89.7% for households with more than 8 members. The percentage of households with at least one net for every two people was 73.1% for households with one member and 28.2% for households with more than 8 members. The percentage of people with access to a net in households with one member was 73.1% but in those of more than 8 members it was 66.5% (Fig 1)

Small but significant differences in LLIN ownership and access were observed across household deprivation levels (χ^2^, df = 2, p<0.0001 in all tests). Wealthiest households presented better LLIN ownership and access levels. In 2015, optimal ownership was 3.9% higher and household member access to a net was 5.4% higher in the wealthiest households compared to the poorest ones. These differences increased in 2016, when optimal ownership was 11.5% higher and member access to a net was 11.2% higher in the wealthiest households, compared to the poorest ones (S1 Table).

There were also significant differences in LLIN ownership and access across administration subdivisions of the district (χ^2^, df = 4, p<0.0001 in all tests). In 2015 and 2016, the lowest percentage of households that owned at least one LLIN was observed in Panjane (77.9% and 74.3%, respectively) with the highest values observed in Motaze (90.8% and 84.3%, respectively). Likewise, the lowest percentage of households with at least one net for every two people was observed in Panjane (51.5% and 43.2%, respectively) and the highest values in Motaze (72.0% and 58.5%, respectively). Residents’ access to sleeping under an LLIN was lowest in Mapulanguene (71.3% and 61.6%, respectively) and highest again in Motaze (82.9% and 73.1%, respectively) (S1 Table)

**Fig 2:**
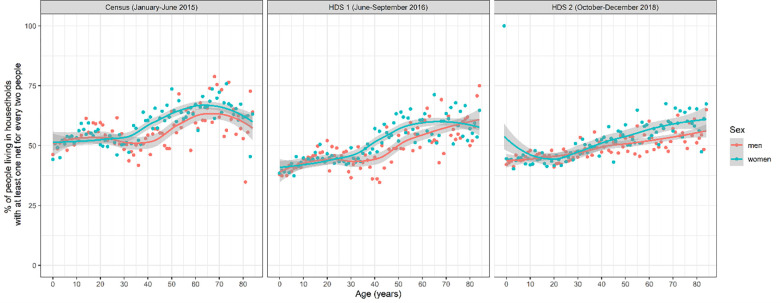
Percentage of people living in households with at least one LLIN for every two people, segregated by gender and age. Grey shaded areas represent the locally estimated scatterplot smoothing of the curves.

The percentage of people that lived in a household with at least on net for every two people varied with age and sex. It was lowest in the youngest age groups, increased among school-aged children and young adults, decreased again in adults between 20 and 40 years of age, and subsequently increased in older ages. In those over 30 years of age, it was higher among women than men, but in those below 30 years of age it was similar across sexes (Fig 2).

### LLIN use

Out of the 24,302 nets for which information was collected during the 2015 population census (97% of all nets in the district), 47.9% had been used the night before, 51.2% had not been used the night before and 0.8% of the respondents did not know if the net was used the night before.

Between January and June 2015 (before the project started) 25.4% of residents reported sleeping under a net. During the project, individual LLIN use varied seasonally, with a maximum LLIN use of 76.4% (76.0–76.8) in January 2016 (MDA data, high transmission season) and a minimum of 40% in June-August 2016 (DHS data, low transmission season) (Table 2). Individuals living in households with at least one net for every two people were more likely to sleep under a net than those sleeping in households with less than one net for every two persons (χ^2^, p<0.001, for all years) (Table 2)

LLIN use varied between administrative posts (S2 Table). Use was generally higher in Magude Sede (the district’s capital) and Motaze (the second most urbanized locality) than in the more rural areas of Magude (χ^2^, p<0.001). Use was higher in the richest households than in the poorest ones, with differences up to 14% (χ^2^, p<0.001) and higher in smaller households than in larger ones before the project started, with difference up to 14.5% (χ^2^, p<0.001), but similar across household sizes during project itself.

**Fig 3:**
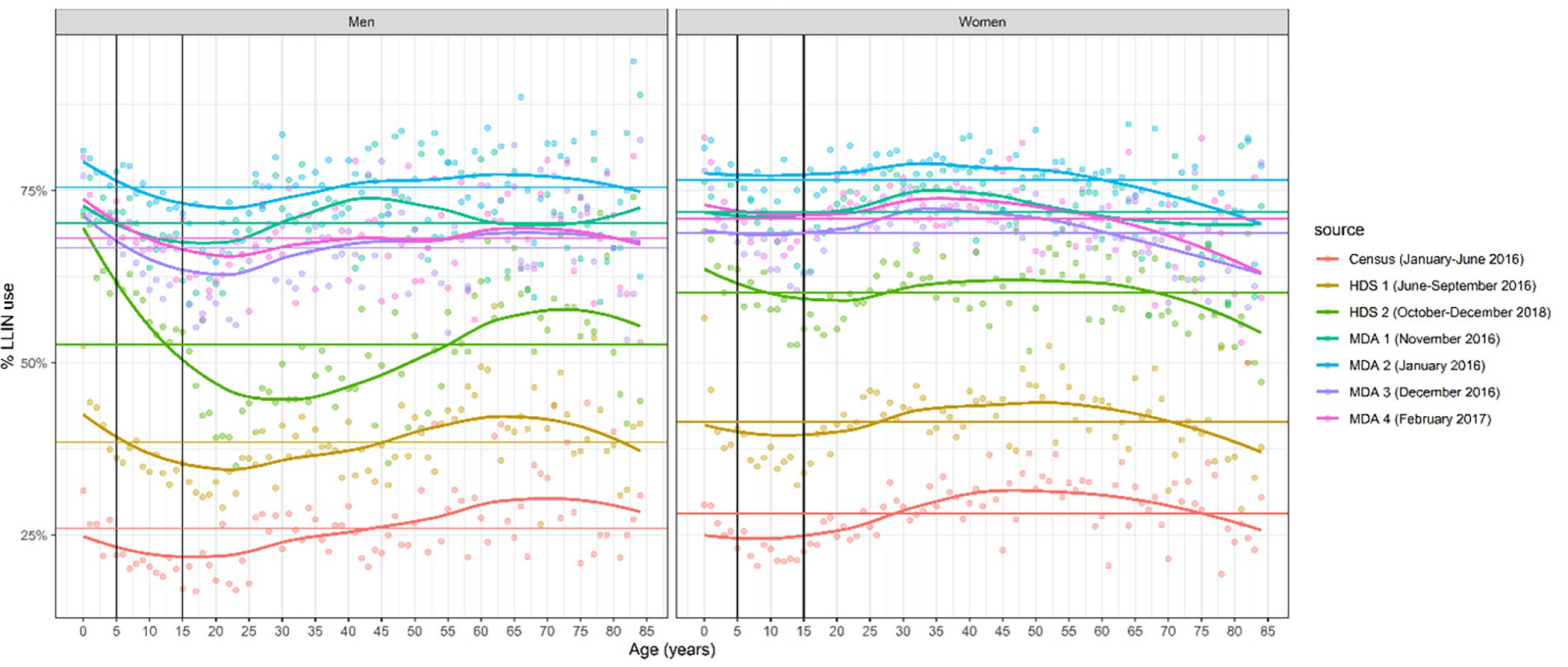
LLIN use across age and gender in Magude district during the project. Each line represents data from a single survey or study conducted during the Magude project. Each point is the average bednet use found for the corresponding age in the corresponding study. MDA estimates represent between 77.7% and 89.6% of the population, depending on the MDA survey.

LLIN use varied considerably with age and gender (Fig 3). Differences between men and women were more accentuated during the low transmission months. Among men, LLIN use was generally highest in children under 5 years of age, was especially low in men between the ages of 18 and 30, increased steadily until the age of 65 and 75 to decline again in older men (Fig 3). The same pattern was observed in both transmission seasons, although differences across age groups were more accentuated in the low transmission season. Among women, LLIN use was generally higher in women between the age of 30 and 55 than in women of other ages. During the high transmission season, net use was lowest in women of older ages and during the low transmission season in female adolescents until the age of 20 (Fig 3). As observed from the census of the population of 2015 and health and demographic survey of 2016 and 2018 (Table 2), pregnant women were more likely to sleep under a net that non-pregnant women (χ^2^_,_ p = 0.001), although the difference was not large.

**Fig 4:**
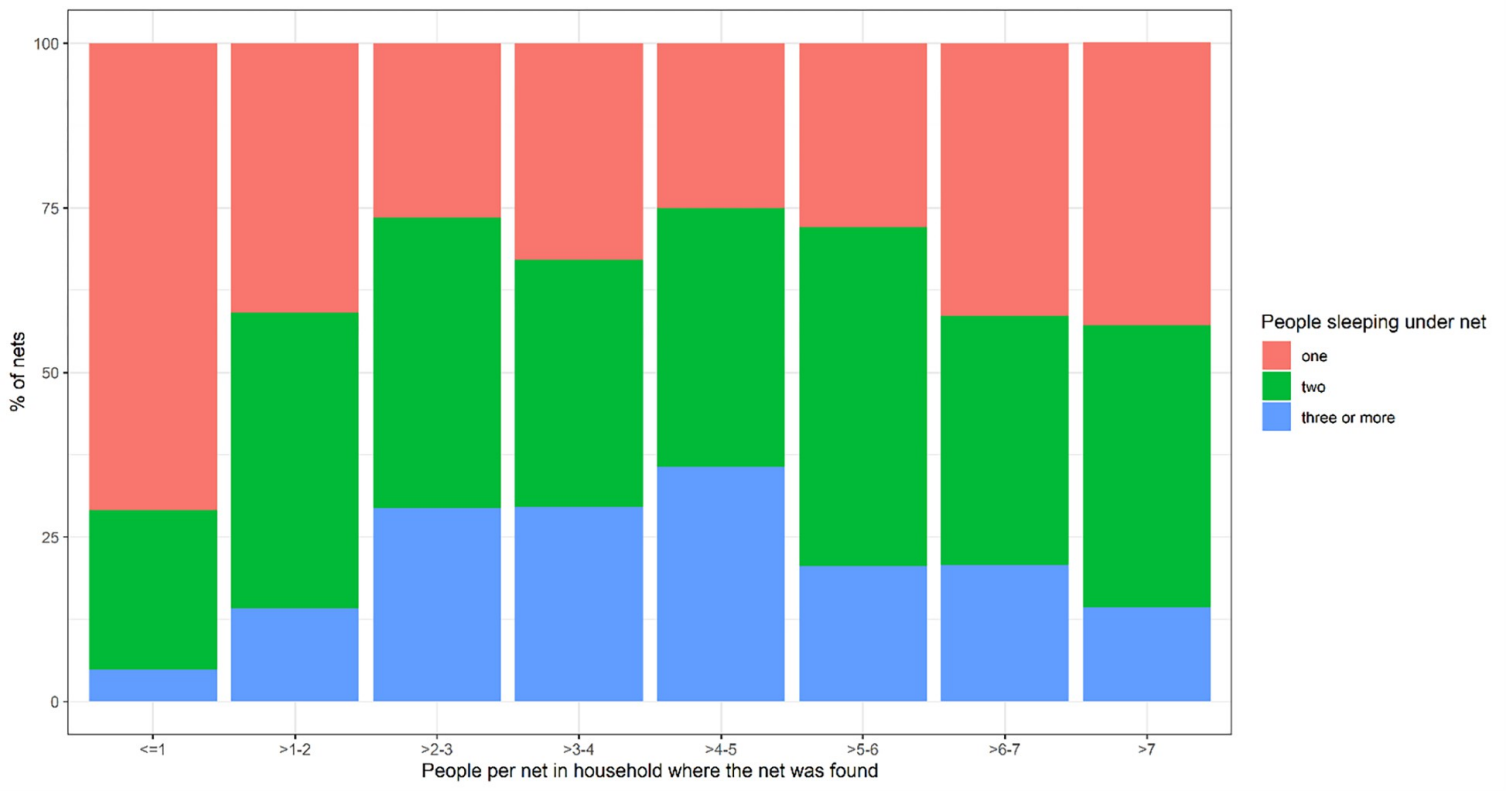
Number of people sleeping under a net according to the number of people per net in the household where the net was found.

At the time of the population census conducted at the beginning of the project (January- June 2015), the number of people per net was 1.7 (SD: 0.8) with a median of 2 (IQR 1–2), and 47.5% of nets found in all households (n = 24,302) were used by one person, 38.5% by two people and 14.1% by three or more people. The percentage of nets used by three or more people increased with the number of people per net in the household up to 5 people per net and decreased thereafter (Fig 4).

### Reasons for not using an LLIN to sleep

The main reasons for not using an LLIN (data from MDA surveys conducted during the high transmission seasons) was not having a net (56.3% [55.4–57.2], 61.8% [60.8–62.8], 67.1% [66.3–68.0] and 77.6% [76.9–78.4] in November 2015, January 2016, December 2016 and February 2017, respectively), followed by the bednet not being hung (22.6%[21.8–23.2], 23.3%[22.4–24.2], 18.8%[18.2–19.5] and 14.7% [14.1–15.3], respectively), disliking of the bednet (10.9% [10.3–11.5], 8.3% [7.8–8.9], 8.2[7.7–8.7] and 5.0% [4.6–5.4], respectively) or that it was too hot (7.8% [7.3–8.3], 5.5% [5.1–6.0], 4.4% [4.1–4.9] and 1.4% [1.3–1.7], respectively). The proportion of individuals who claimed not using the net due to not having one increased over time across the four MDA rounds.

### Type of nets used by residents

In May 2015, 2016 and 2017, 79.0% (65.8–88.0), 91.0% (88.8–93.0) and 91.2% (89.6–93.0) of respondents, respectively, used a net that was impregnated during the last 12 months or a long-lasting insecticidal net the night before the interview (data from the malaria prevalence cross sectional surveys). In May 2015, 2016, 2017 and 2018, 95.5% (84.7–99), 81.4% (78.4–84), 85.1% (83.2–87) and 96.5% (95.1–98) of respondents, respectively, reported to have received the net they used from a health facility or the NMCP and 0.4% (0.5–3), 3.4.% (2.3–5), 2.9% (2.1–4) and 0.7% (0.3–2), respectively, reported to have bought the net. In the population census conducted in 2015, the majority of the 24,302 nets for which information could be collected (97% of all nets in the district), were Olyset® Nets (77.1%), followed by Permanet® 2.0 (21.1%), Netprotect® (0.5%), Interceptor® (0.5%), Duranet® (0.1%) and DawaPlus® (0.1%), and of 0.9% the brand was unknown.

## Discussion

The present study evaluated LLIN ownership, access and use during the Magude project and inequalities in these indicators across district subdivisions, household sizes, household wealth, and individuals’ sex and age. Such information is critical to improve our understanding of the protection that LLINs actually confer during malaria control and elimination programmes, including the Magude project, and to identify ways to improve LLIN access and use.

Most nets that were found in Magude district during the project were obtained from the NCMP or a health facility (>88%), suggesting they were received during the LLIN mass distribution campaign, or through the ante-natal care services (ANC) or expanded programs of immunization (EPI). Less than 3.4% of the nets had been bought. From the start of the project up to December 2017 (when the next LLIN mass distribution campaign took place) most residents slept under an Olyset® Net, as this brand accounted for 77.1% of the nets identified in the district.

The percentage of households that owned at least one net ranged from 78.8% to 91.1%, suggesting that there were gaps in household coverage shortly after the mass distribution campaigns. Household’s optimal LLIN ownership and individual access ranged from 54.4% to 59.2% and from 68.2% to76.3%, respectively, during the project. The percentage of households with optimal LLIN ownership and the percentage of people with access decreased from 61.9% and 73.7% at the start of the project to 54.4% and 68.2% during the second year of the project, respectively. Despite the distribution of over 25% more nets in the 2017 campaign compared to 2014 campaign, optimal LLIN ownership and individual access in 2018 increased but remained at levels similar to those measured at the beginning of the project, 59.2% and 76.3%, respectively. Given that the rate of net loss after both campaigns was similar (approx. 31% during the first year), this finding suggests that the distribution of larger quantities of nets in 2017 did not improve LLIN ownership or access. This could be due to an inadequate distribution of nets. Indeed, we observed inequalities in LLIN optimal ownership and access during the project, with larger households and those located in more remote areas being more frequently underserved than wealthier households or households located in easier-to-reach areas. This suggests that the households that are missed during the campaigns may have been those that are located in harder-to-reach areas and that LLIN allocation strategies during the campaign did not adequately cover the needs of larger households. Combined with the fact that larger households showed lower LLIN survival over time elsewhere in Mozambique [23], the protective efficacy of LLINs in larger households may even have been lower in Magude district. Inequalities were also observed by household wealth, with wealthier households owning more nets, which has been observed in other countries [24, 25]. These inequalities exacerbated over time after the mass distribution campaign. Although we did not investigate the reasons, this may be due to poorer households using the nets for other purposes than sleeping (e.g. fishing or the protection of fruits and seedlings [26]) or due to a more rapid deterioration and subsequent disposal of nets because of e.g. poorer storage conditions or net care practices [27]. This should be assessed in greater detail, as a more rapid net loss in poorer households leads to a greater gap in protection by LLINs.

LLIN attrition rates in Magude were higher than those reported in other provinces in Mozambique (Tete, Nampula and Inhambane) after the mass distribution campaign in 2017 [23]. Although we did not assess the reasons for net loss, the most frequently reported reason in the other provinces included throwing the net away, that the net was destroyed or that it was used for other purposes [23]. The rapid loss of LLINs reduced LLIN access and compromised the protection that LLINs could have provided throughout the Magude project. Such rapid loss suggests that a more frequent distribution of LLINs and/or improving community practices to ensure the survival of nets over time would be necessary to ensure high access over time. Although currently not recommended by the World Health Organization (WHO) for programmatic settings, top-up campaigns could have been implemented in Magude district to compensate for LLIN attrition and the inequalities in net ownership as the necessary data for decision-making were recorded through the annual health and demographic surveys.

Surprisingly, even though LLINs were reportedly being distributed during antenatal care services throughout the Magude project, access was lower among children than among adults and lower in women below 30 than in older ages. This could be due to the combination of low use of immunization or antenatal care services (between 25% and 31% of pregnant women never used antenatal care services [16]), as well as to LLIN stock-outs in health facilities, and should be further investigated.

The majority of Magude residents who slept under a net before the project started either used a net that was impregnated over the last 12 months or a LLIN (79% in 2015 and >91% in other years), which suggests that LLINs could have provided both personal protection (i.e. reducing vector-host contact) and contributed towards reducing population densities of local pyrethroid-susceptible vectors in the district. LLIN use increased throughout the project from a baseline level of 25.4% in January-June 2015 to 64.4% and 76.3% in January and May 2016, respectively, and to 70.3% and 72.2% in February and May 2018, respectively. Since the percentage of households with optimal LLIN ownership and the percentage of people with access to a net did not increase during the project, the observed increase in LLIN use is likely due to community engagement campaigns that were implemented during the Magude project. This suggests that reaching the target of 2 people per net through mass distribution campaigns would not have sufficed to guarantee high levels of LLIN use in Magude. Although the average number of people per net at the beginning of the project (~1 year after distribution) was 1.7, with a median value of 2, more than 25% of the nets located in households with more than two people per net were used by a single individual. This shows the need to revise the allocation strategies during mass distribution campaigns, or to promote net sharing by two individuals during community engagement campaigns.

LLIN use varied seasonally, reaching a maximum of 76.3% in the high transmission months but being as low as 40% during the low transmission season. During the high transmission seasons, LLIN use seems to have been limited by LLIN access, as the most frequently reported reason for not using a net to sleep was not having one, and the percentage of participants reporting this reason increased over time as LLIN access decreased. The fact that LLIN use exceeded LLIN access at times during this season shows the population’s willingness to use a net during this season. This suggests that increasing LLIN access in Magude would have increased LLIN use during the high transmission season. This could have further reduced malaria transmission. In contrast, during the low transmission season, LLIN use was highly limited by human willingness to use their net, in addition to gaps in access. In this season, the percentage of people sleeping under a net among those living in households that had at least one net for every two people was only 55.1% (June-August 2016). Seasonal variations in LLIN use have been observed in several other countries and have been commonly linked with vector abundance and/or heat [12, 28–32]. Raising awareness of the risk of contracting malaria during this season is critical to increase LLIN use. This is especially important in Magude, as a significant proportion of malaria transmission during the Magude project occurred during this season [16].

As seen in other African settings [33–37], LLIN use was lowest in school-age children and young adults, especially among young males, highest in children under 5 and in older adults, and in general higher among women than among men. Since young adults (5–15 years old) have been observed to act as important reservoir of malaria parasites in neighboring countries [38, 39], and the infection rates in Magude residents of >5 years of age were similar or -at times- higher than in those <5 yo [16], the low levels of net use in this age group may have contributed toward sustaining malaria transmission during the Magude project. The variation of LLIN use with age also suggests that the common disaggregation of LLIN use in the three age groups recommended by WHO (under 5, 5–15 and >15 years of age) [20] may not accurately reflect the age-related differences in net use. It further highlights the importance of implementing behavior change activities targeting specific age groups, especially young males.

LLIN use was slightly higher among people living in sprayed households (i.e. covered by IRS) than among those living in unsprayed households. Although the reasons were not evaluated in the present study and the number of unsprayed households was very low, this suggests that deploying IRS in combination with LLINs may have had a positive impact on LLIN use. Such synergistic associations has been previously observed in Magude [16] and elsewhere [40, 41], which highlights the potential added value of deploying the two interventions together.

At the start of the project, 14% of nets were used by three or more people. Net sharing among more than two individuals likely continued during the project as LLIN use exceeded LLIN access at specific points in time. The downside of sharing a net with more than 2 persons is that this can reduce the protection provided by LLINs as people’s limbs can be against or stick out from underneath the net due to limited space availability, allowing mosquitoes to feed on net user(s). A study conducted in Kenya showed that malaria prevalence in children who slept with two or more additional people under a net was similar to that in children that did not use a net to sleep [42]. In Guinee Bissau, a similar trend was seen in hospital visits by children [43]. As such, net sharing preferences should be taken into consideration during mass distribution campaigns, either by distributing larger nets to households where more than two people share a net (e.g. due to limited sleeping space) and/or by raising community awareness on best LLIN use practices through communication campaigns.

This study draws from different surveys to report LLIN ownership, access and use at different time points. This provides valuable insights into how access decreases after LLIN mass distribution campaigns, how inequalities in LLIN ownerships and access evolve over time and how seasonality affects LLIN use. The present study has several limitations, as the surveys were not specifically designed to measure LLIN indicators. The first limitation is that MDA surveys represented between 77.7% and 89.6% of the Magude population, and as such our point estimates do not represent the entire population of the district. Nonetheless, by estimating the best-case and worst-case scenario intervals, a representative range of confidence in the values is provided. Second, attrition rates were evaluated retrospectively, and may have been underestimated as some nets were likely received through ANC and EPI services rather than through the LLIN mass distribution campaign. Finally, the reasons for net loss, and for not using a net during the low transmission season were not quantified, which is critical information to guide future behavioral change campaigns aimed at improving the impact of LLINs.

## Conclusion

LLIN ownership, access and use were heterogeneous and sub-optimal during the Magude project. People living in hard-to-reach areas, poorer and larger households, and young males were associated with poorer LLIN access and lower LLIN use during the project. The combination of LLINs and IRS had a positive effect on LLIN use. Mass-distribution campaigns alone were not enough to achieve the high level of LLIN protection needed during a malaria elimination program. To ensure high and equal levels of LLIN protection, future mass LLIN campaigns in Mozambique and elsewhere, especially in elimination settings, should a) revise LLIN allocation scheme to ensure equal LLIN ownership and access across all households and population groups, b) consider LLIN top-up campaigns to fill the gaps in LLIN access resulting from LLIN allocation schemes and LLIN attrition post-campaign, and c) implement behavioral change campaigns to ensure high LLIN use, especially during the low transmission season, among school-aged children and young males in harder-to-reach areas and in the poorest households. Further research is needed to investigate the reasons for: 1) current net allocation strategies leading to inequalities in bednet ownership and access, 2) the poor LLIN use observed during the low transmission season, 3) the low use observed in young males, 4) the faster net loss observed in Magude compare to other districts in Mozambique, and 5) LLIN access being lower in children under 5 and pregnant women despite continuous LLIN distribution through ANC and EPI.

## Supporting information

S1 Table LLIN ownerships and access by locality and wealth index in Magude district. (DOCX)

S2 Table LLIN use by administrative post in Magude district. Data sources are the same as for Table 1 in the main manuscript.
(DOCX)
