# Supplementary material for: An evaluation of LLIN ownership, access, and use during the Magude project in southern Mozambique
Source: PLoS One. 2023 Mar 27;18(3):e0282209. doi: 10.1371/journal.pone.0282209 (PMC10042371; doi:10.1371/journal.pone.0282209)
Supplement: S1 Table — (DOCX) [file pone.0282209.s001.docx]

**S1 Table. LLIN ownerships and access by locality and wealth index in Magude district**

|  | % of HH with at least 1 net | | % of HH with at least 1 net for every 2 people | | % of people with access to an LLIN within their household | |
| --- | --- | --- | --- | --- | --- | --- |
| By locality | 2015 | 2016 | 2015 | 2016 | 2015 | 2016 |
| - Magude Sede | 80.1 | 79.2 | 61.1 | 55.6 | 72.4 | 68.8 |
| - Motaze | 90.8 | 84.3 | 72.0 | 58.5 | 82.9 | 73.1 |
| - Panjane | 77.9 | 74.3 | 51.5 | 43.2 | 68.7 | 60.3 |
| - Mahele | 86.1 | 72.6 | 61.5 | 48.3 | 75.8 | 62.8 |
| - Mapulanguene | 78.4 | 70.7 | 58.4 | 45.1 | 71.3 | 61.6 |
| By wealth index (MPI)* | 2015 | 2016 | 2015 | 2016 | 2015 | 2016 |
| 0 | 83.0 | 85.7 | 63.9 | 59.9 | 75.0 | 74.2 |
| 1 | 82.9 | 80.0 | 61.4 | 53.1 | 74.3 | 67.7 |
| 2 | 77.4 | 74.2 | 60.0 | 52.6 | 69.6 | 63.0 |

** The MPI was reclassified into three categories according the number of deprivations: Category 1 (0-2); Category 2 (3-4); Category 3 (5-6). The higher the number of deprivations, the poorer the household.*
