# Supplementary material for: An evaluation of LLIN ownership, access, and use during the Magude project in southern Mozambique
Source: PLoS One. 2023 Mar 27;18(3):e0282209. doi: 10.1371/journal.pone.0282209 (PMC10042371; doi:10.1371/journal.pone.0282209)
Supplement: S2 Table — Data sources are the same as for Table 1 in the main manuscript. (DOCX) [file pone.0282209.s002.docx]

**S2 Table.** **LLIN use by administrative post in Magude district. Data sources are the same as for Table 1 in the main manuscript.**

| By locality | Magude | Motaze | Panjane | Mahele | Mapulanguene |
| --- | --- | --- | --- | --- | --- |
| 2015 (Jan-Jun) | 27.2 | 27.3 | 14.3 | 16.0 | 11.5 |
| 2015 (November) | 70.7 | 79.1 | 62.3 | 73.0 | 58.3 |
| 2016 (January) | 75.7 | 85.0 | 70.3 | 78.7 | 69.7 |
| 2016 (Jun-Aug) | 42.6 | 41.4 | 30.2 | 30.9 | 20.4 |
| 2016 (December) | 68.3 | 73.3 | 60.7 | 57.0 | 71.1 |
| 2017 (February) | 70.3 | 75.8 | 64.1 | 67.6 | 60.9 |
